# Clinical Outcomes of Adults with Systemic Mastocytosis: A 15-Year Multidisciplinary Experience

**DOI:** 10.3390/cancers14163942

**Published:** 2022-08-16

**Authors:** Johanna Ungerstedt, Christopher Ljung, Monika Klimkowska, Theo Gülen

**Affiliations:** 1 HERM Hematology and Regenerative Center, Department of Medicine Huddinge Karolinska Institutet, SE-14183 Stockholm, Sweden; 2 ME Hematology, Karolinska University Hospital, SE-14186 Stockholm, Sweden; 3 Department of Respiratory Medicine and Allergy, Karolinska University Hospital Huddinge, SE-14186 Stockholm, Sweden; 4 Pathology Unit, Department of Laboratory Medicine, Karolinska Institutet, SE-14183 Huddinge, Sweden; 5 Department of Clinical Pathology and Cytology, Karolinska University Hospital, SE-14186 Huddinge, Sweden; 6 Department of Medicine Solna, Karolinska Institutet, SE-17177 Stockholm, Sweden; 7 Department of Medicine Huddinge, Karolinska Institutet, SE-14183 Stockholm, Sweden

**Keywords:** mastocytosis, overall survival, prevalence, incidence, multidisciplinary approach, KIT D816V, tryptase, β2-microglobulin, SM-AHN, aggressive SM

## Abstract

**Simple Summary:**

The term mastocytosis denotes a heterogeneous group of rare disorders characterized by abnormal accumulation and activation of mast cells in various organs. Based on the WHO classification, mastocytosis can essentially be split into indolent and advanced systemic disease. Indolent mastocytosis is the most prevalent variant in adults. In our retrospective study, we aimed to analyze the clinical outcomes of patients with mastocytosis attending our center over the last 15 years. We found that most patients in our cohort suffered from the indolent variant of mastocytosis. Furthermore, we determined the overall prevalence of mastocytosis in our region to be one case per 10,000 adults. In addition, overall survival was significantly better in indolent mastocytosis patients compared to patients with advanced disease. Therefore, awareness of differences in the prognoses of patients with indolent and advanced mastocytosis is important.

**Abstract:**

Systemic mastocytosis (SM) is a rare, clonal, clinically heterogeneous disorder of the mast cells (MCs), and mainly affects adults. The present study aims to describe the clinical and laboratory features as well as the outcomes of SM. A 15-year retrospective study was conducted on 195 consecutive SM patients (aged ≥ 18 years) diagnosed in 2006–2020 at the Multidisciplinary Mastocytosis Center at Karolinska University Hospital. Patients with indolent SM (ISM) represented the most common SM variant (88.2%). Furthermore, the frequencies of aggressive SM and SM with associated non-mast-cell hematological neoplasm were 4.1% and 7.7%, respectively. The prevalence of SM in the adult population of the Stockholm region was estimated to be 10.6/100,000 inhabitants, and the mean incidence of SM cases in the Stockholm region was 0.77/100,000 people per year. In this series, tryptase levels were below 20 ng/mL in 51 patients (26%). Osteoporosis was present in 21.9% of all cases. Interestingly, there was no progression from ISM to advanced SM variants in our study. Furthermore, overall survival was significantly better in ISM patients compared to advanced SM patients (*p* < 0.0001). Our data suggest that the early recognition and correct diagnosis of SM has prognostic significance.

## 1. Introduction

Systemic mastocytosis (SM) is a rare, clonal disease of the mast cells (MCs) [1,2]. Its clinical presentation is extremely heterogeneous, where the symptoms are caused by activation of MCs as well as accumulation of pathological MCs in various organs—most frequently the skin, bone marrow, liver, spleen, and gastrointestinal tract mucosa [1,2].

Mastocytosis is classified into different variants, where SM mainly affects adults, whereas cutaneous mastocytosis (CM)—in which accumulation of MCs is limited to the skin—is by far the most common variant in pediatric patients [1,2]. The histopathological evaluation of bone marrow (BM) is crucial for the diagnosis, as the BM is the most commonly involved extracutaneous organ [1,2]. One major and four minor criteria have been defined for the diagnosis of SM, and according to the World Health Organization (WHO) criteria, the diagnosis of SM can be established by the presence of the major criterion together with one minor criterion or, alternatively, the presence of at least three minor criteria [1,2,3].

The majority of patients have indolent SM (ISM), which generally has a mild course and does not affect overall survival. However, it is increasingly recognized that ISM patients with or without skin lesions have clinically distinct phenotypes, and the newly designated provisional WHO subvariant bone marrow mastocytosis (BMM) covers ISM patients without skin lesions [3,4,5]. Smoldering SM (SSM) is another subtype, and is defined by the presence of organ involvement without organ dysfunction (B-findings). SM with an associated hematological neoplasm (SM-AHN) is the second most common form of SM. In 85–90% of cases, SM-AHN is associated with a myeloid disease (e.g., myelodysplastic/myeloproliferative neoplasms, acute myeloid leukemia) [6], or more rarely with myeloma or lymphoma [7,8]. In SM-AHN, the prognosis is largely dependent on the associated hematological neoplasm. Furthermore, aggressive SM (ASM) is characterized by organ dysfunction due to infiltration of MCs (C-findings). This subtype frequently needs cytoreductive therapy and/or KIT-targeting tyrosine kinase inhibitors (TKI). Patients with SM-AHN and ASM are together commonly denoted as advanced SM (AdvSM), and these patients, in contrast to those with indolent SM, have shortened life expectancy. There are also rare cases of mast cell leukemia (MCL).

The estimated prevalence of SM in adults is approximately 10–13 in 100,000 residents in European countries [9,10,11,12], and patients with SM are known to have a wide diversity of symptoms. These can vary from the typical cutaneous symptoms, such as flushing and itching, to other organ specific symptoms such as osteoporosis, diarrhea, or unexplained syncope [1]. Most symptoms are caused by the release of MC mediators—mainly histamines and pro-inflammatory cytokines [1]; thus, life-threatening anaphylaxis is the classic feature of patients with SM, and particularly ISM [13]. Due to its heterogeneous presentation, diagnosis of SM can be a true challenge, and requires high clinical competence. Any delay in the diagnostic process can lead to several undesired consequences, such as organ dysfunction, life-threatening anaphylaxis, or severe osteoporosis [14,15].

In the present study, we describe the clinical and laboratory characteristics as well as the survival outcomes of patients with SM who were diagnosed and followed up at the Mastocytosis Center of Karolinska University Hospital over the last 15 years, applying a multidisciplinary approach with pathology, allergology, hematology, immunology, endocrinology, gastroenterology, and psychiatry specialists to ensure proper diagnostics and follow-up of each patient. Furthermore, we sought to investigate the prevalence and incidence of SM in our region. As we have previously reported on the allergological features of SM patients in detail [13,16,17,18,19,20], in the present study we aim to create a better understanding of the ways in which patients present to us, and consequently improve the diagnostic and follow-up processes.

## 2. Methods

### 2.1. Patients

The Center of Excellence for SM at Karolinska was established in January 2006, and by December 2020, a total of 349 consecutive adult patients (aged ≥ 18 years) were referred to the center because of clinically suspected mastocytosis. All patients underwent medical evaluation, including BM investigation, to determine the existence of a potential clonal MC disease. Diagnosis of SM was carried by a complete clinical and physical workup together with routine laboratory chemistry and differential peripheral blood count. All patients were seen by an allergist and a hematologist, and in some cases even by a dermatologist, endocrinologist, psychiatrist, or gastroenterologist. MCs in BM biopsy samples were evaluated following previously established methods and criteria for morphology, histology, and immunohistochemistry, along with flow cytometry [21] and mutational analysis [22]. Blood samples for assay of baseline serum tryptase (sBT) (Thermo Fisher^®^, Uppsala, Sweden) were drawn either on the day of BM biopsy or the nearest possible day, but never at the time of anaphylactic reactions. Of the 349 investigated patients, a diagnosis of SM was established in 216 patients, using the current WHO criteria [1,2].

Patients with a confirmed SM diagnosis underwent routine investigations, including ultrasound or computerized tomography of the abdomen to detect B- and C-findings and to diagnose advanced variants of SM, and their bone mineral density (BMD) was measured by dual X-ray absorptiometry at the lumbar spine and proximal hip to identify patients with bone involvement. The presence of osteoporosis was defined according to the WHO criteria, as a BMD T-score of ≤−2.5 SD [23]. As the SM patients included premenopausal females and males aged <50 years, we also used a Z-score threshold of −2.0 SD as a diagnostic criterion in these patients. A pathological fracture was defined as a spontaneous fracture directly linked to SM. Additionally, osteosclerosis was classified according to T-scores ≥ 2.5.

Patients were routinely seen annually or biennially for follow-up purposes in our outpatient clinics (mainly in allergy and/or hematology, but in some cases—such as severe osteoporosis—also by endocrinology), and data on clinical symptoms, along with information on their progression/survival, were retrieved.

### 2.2. Study Design

We conducted a retrospective study of the patients diagnosed with SM at our Center. Of the 216 SM patients, 195 provided their written consent and were enrolled in the study. The study was approved by Stockholm’s Ethics Review Board (Dnr: 2011/1750/31/3 and Dnr: 2018/2621-31). The medical records of the 195 patients—including laboratory test results, imaging results, and pathological analyses of biopsy materials—were reviewed and collected from the electronic patient charts.

Patients were censored at death or at last follow-up (30 November 2020)—whichever came first—and overall survival and possible risk factors of death were analyzed.

### 2.3. Statistical Analysis

All analyses were performed using R (R version 3.6.1., Vienna University of Economics and Business, Vienna, Austria). All tests were 2-sided, and *p*-values < 0.05 were considered statistically significant. Categorical variables were presented as absolute numbers and percentages, and analyzed using the Chi-square test or Fisher’s exact test, when appropriate. Continuous variables were presented as median values and ranges. Because the distribution of the data was not normal, nonparametric tests were used to compare the group means. When testing group mean differences in variants of SM (more than 2 groups), the Kruskal–Wallis test was used. The associations between BM MC infiltration and beta-2-microglobulin (B2M), alkaline phosphatase (ALP), and sBT were assessed using Spearman’s rank correlation test.

Moreover, markers reported to be correlated with disease severity—namely, B2M, ALP, and serum tryptase—were studied in relation to the presence/absence of major criteria, and to vital status; *p*-values were calculated using the Mann–Whitney *U*-test.

The probability of survival was estimated using the Kaplan–Meier estimator. The comparison between survival curves was evaluated using the log-rank test. Because the differences in overall survival (OS) between interventional groups (ISM vs. advanced SM) were confounded by age, we created an age-matched cohort by using the propensity score matching (PSM) method to minimize selection bias [24]. In a subsequent step, we used univariable and multivariable Cox regression models, expressed as hazard ratios (HRs) with 95% confidence intervals (CIs), to estimate the HR of mortality for several variables of interest.

Furthermore, the prevalence of SM was estimated as the proportional relationship between the number of affected patients and the total adult inhabitants residing in the Stockholm region on 31 December 2020. The total number of adult (aged ≥ 18 years) inhabitants was reported to be 1,817,912 (76% of the total 2,391,990) at this time in the Stockholm region. Likewise, we estimated the mean incidence of new cases per 100,000 people per year over the follow-up period, as the population size in the Stockholm region did not substantially change over the several years (about 16% increase between 2007 and 2018). Data on the resident population were taken from the website of the Swedish Central Bureau of Statistics, https://www.scb.se/hitta-statistik/statistik-efter-amne/befolkning/befolkningens-sammansattning/befolkningsstatistik/ (accessed on 22 February 2021).

## 3. Results

### 3.1. Patient Characteristics

In our series, a total of 172 patients obtained the diagnosis of ISM (including 3 patients with SSM) (88.2%), while 8 had ASM (4.1%), and 15 had SM-AHN (7.7%) (Table 1). There were no cases of MCL in this cohort. The median age at diagnosis of SM was 57 years; however, ISM patients were significantly younger at diagnosis when compared to patients with AdvSM (*p* < 0.001). Among the patients, females were slightly over-represented (55.4%), although this did not reach the level of statistical significance. Table 1 shows the detailed characteristics of the patients. However, when we separately analyzed ISM patients with or without the major criterion, there was a significantly higher frequency of females among ISM patients without the major criterion (*p* = 0.034) (Supplementary Table S1). Overall, typical mastocytosis skin lesions occurred in 68% of patients, and more commonly in ISM patients compared to patients with AdvSM (*p* < 0.001). Of the ISM patients, 47 had no typical skin lesions (27.8%) and obtained the diagnosis of BMM. Moreover, as shown in Supplementary Table S2, three patients had B-findings and eight patients presented with C-findings, and therefore were diagnosed with SSM and ASM, respectively.

The median time from the occurrence of the first patient-reported symptoms to the diagnosis of SM was 10 years (range, 0.5–47 years). Furthermore, the prevalence of SM in the adult population (aged ≥ 18 years) of the Stockholm region was estimated to be 10.6 per 100,000 inhabitants during the study period. Likewise, we roughly estimated the mean incidence of new SM diagnoses/year in the Stockholm region, and found it to be 0.77 per 100,000 people per year.

Comorbidities in the form of solid tumors were documented in 18 patients (9.2%) in this cohort; of these, 5 had cutaneous melanoma (2.5%), 4 had breast cancer, 4 had gastrointestinal malignancies, 2 had pancreatic cancer, 2 had small-cell lung cancer, and 1 had squamous-cell carcinoma.

### 3.2. Laboratory Findings

The median sBT level in the cohort was 32 ng/mL (range 4.3–710). Interestingly, sBT levels were lower than 20 ng/mL in 51 patients (26%), and lower than 10 ng/mL in 15 patients (8%). Furthermore, sBT levels were significantly higher in patients with AdvSM compared to patients with ISM (*p* < 0.001). In addition, sBT levels were significantly higher in ISM patients who had the major criterion compared to ISM without the major criterion (*p* < 0.001) (Supplementary Table S1).

Moreover, B2M and ALP levels measured at diagnosis were significantly higher in patients with AdvSM (*p* < 0.001 and *p* < 0.001, respectively) (Table 1). Likewise, elevated levels of eosinophils as well as monocytes were mainly observed in AdvSM patients (*p* = 0.023 and *p* = 0.037, respectively); however, these results were not statistically significant after the adjustment of *p*-values (*p* = 0.42 and *p* = 0.55, respectively) (Table 1). Furthermore, in 116 cases, zinc levels were available, with a mean as well as median level of 11 μmol/L (reference values 8.0–14 μmol/L). There were no significant differences in zinc levels between different variants of SM, nor in SM patients with or without skin engagement.

### 3.3. Bone Marrow Findings

The major criterion defined by the WHO was fulfilled in 57.4% of cases, and less frequently in ISM compared to AdvSM; however, this was not statistically significant (Table 1). Moreover, atypical morphology (>25%) of MCs on BM smears was detected in 92% of patients, without significant differences between SM variants. Likewise, 96% of patients in the cohort had a *KIT* D816V mutation (Table 1). Regarding 15 patients with SM-AHN, 13 had an associated myeloid neoplasm (6 MDS, 1 MPN, 1 MDS/MPN-U, and 5 CMML), while 2 patients had an associated lymphoid neoplasm (1 Hodgkin lymphoma and 1 follicular lymphoma).

**Figure 1:**
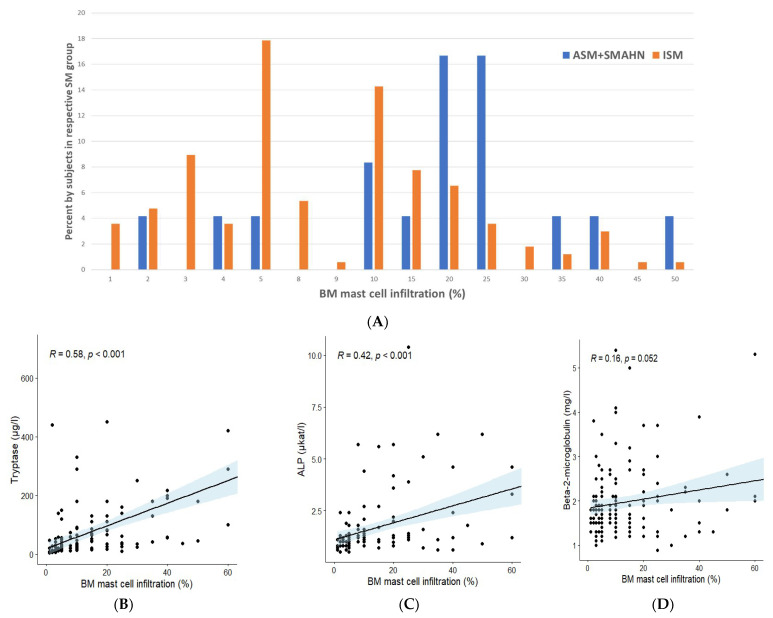
Different aspects of bone marrow mast cell infiltration in different SM variants: (**A**) percentage of bone marrow mast cell infiltration in different SM variants; correlations between bone marrow mast cell infiltration (%) and (**B**) tryptase, (**C**) ALP, and (**D**) B2M were assessed using Spearman’s rank correlation test. Abbreviations: SM, systemic mastocytosis; ALP, alkaline phosphatase; B2M, beta-2-microglobulin.

**Figure 2:**
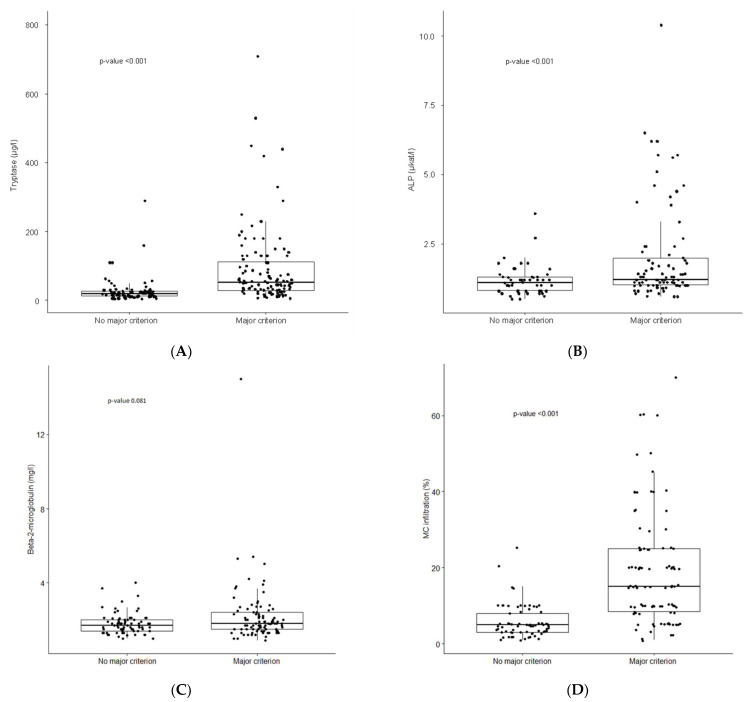
The relationships between the major criterion and tryptase, ALP, and B2M levels: Boxplots are used to explore relationships between the major criterion and (**A**) tryptase, (**B**) ALP, (**C**) B2M, and (**D**) MC infiltration; *p*-values were calculated using the Mann–Whitney *U*-test. Abbreviations: SM, systemic mastocytosis; ALP, alkaline phosphatase; B2M, beta-2-microglobulin.

In addition, we determined percentage of BM MC infiltration in SM variants, and found a significantly higher degree of MC infiltration in patients with AdvSM (mean MC infiltration 24%, median 20%) compared to patients with ISM (mean MC infiltration 11.2% MC, median 8%) (*p* < 0.01) (Figure 1A). Furthermore, we analyzed the relationship between the percentage of MC infiltration and sBT, B2M, and ALP levels. A significant correlation of the percentage of MC infiltration was found with sBT levels (*p* < 0.001) and ALP levels (*p* < 0.001), although not with B2M levels (Figure 1B–D). Likewise, the association between the presence/absence of the major criterion and sBT (Figure 2A), ALP (Figure 2B), and B2M (Figure 2C) levels, along with the percentage of MC infiltration (Figure 2D), was analyzed. SM patients who had the major criterion had significantly higher levels of sBT (*p* < 0.001) and ALP (*p* < 0.001), as well as a higher percentage of MC infiltration in BM (*p* < 0.001), compared to SM patients without the major criterion. Interestingly, however, there were no significant differences in terms of B2M levels (Figure 2C).

In this study, karyotype, target sequencing of potential additional myeloid mutations, and *KIT* D816V mutation allele burden were not routinely assessed.

### 3.4. Bone Mineral Density Abnormalities

Bone mineral density was assessed in 164 patients (84% of the cohort), of whom 36 (21.9%) were diagnosed with osteoporosis (Table 1). The frequencies of osteoporosis in different SM variants were 22.2% in ISM patients, 28.5% in ASM patients, and 20% in SM-AHN patients (Table 1). None of the three SSM patients had osteoporosis. Interestingly, when comparing ISM patients with or without the major criterion, the prevalence of osteoporosis was significantly higher in ISM patients with the major criterion—12% vs. 32%, respectively (*p* = 0.002) (Supplementary Table S1). Fragility fractures were documented in 20 patients (10%) overall, irrespective of osteoporosis status. In addition, we detected seven patients with osteosclerosis, of whom two had SSM, two had ASM and two had SM-AHN. None of the patients with ISM had osteosclerotic lesions.

### 3.5. Therapeutic Strategies: Treatment of Advanced SM

Among 23 patients with AdvSM, the following cytoreductive treatments were used: cladribine (n = 2), alpha-interferon (n = 5), hydroxyurea (n = 6), azacitidine (n = 5), and lymphoma chemotherapy (n = 1). Furthermore, three patients (two with SM-AHN and one with ASM) underwent allogenic hematopoietic stem cell transplantation. Moreover, two patients with ASM were treated with midostaurin.

In this cohort, three patients with SM-AHN did not receive cytoreduction, due to their rapid deterioration. Furthermore, one patient with ASM declined cytoreduction, and one ASM patient did not require cytoreduction.

### 3.6. Clinical Course and Overall Survival

**Figure 3:**
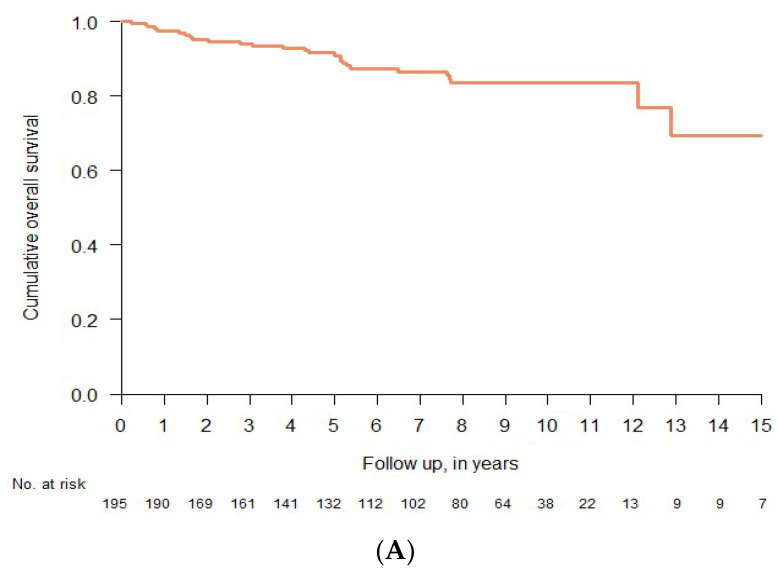

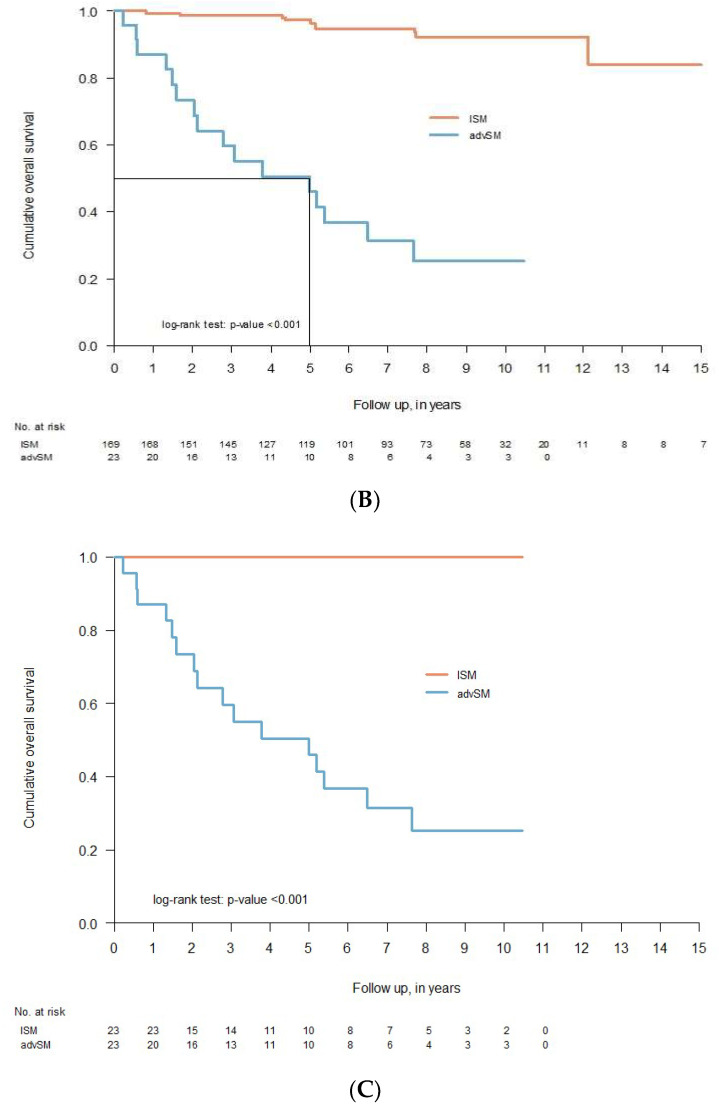
Overall survival of patients with systemic mastocytosis as determined by the log-rank test: Kaplan–Meier estimates of overall survival for (**A**) the full cohort of patients with systemic mastocytosis and (**B**) stratified by ISM vs. AdvSM, and (**C**) age-matched propensity score stratified by ISM vs. AdvSM.

The median follow-up from diagnosis was 7.5 years (range 1.5–21.5). In addition, the 15-year OS for all subjects was 69.3% (95% CI 52.9–90.7) (Figure 3A). OS differed significantly between patients with ISM and patients with AdvSM (*p* < 0.001) (Figure 3B). Among patients with ISM, the 15-year OS was 83.8% (95% CI 69.0–1.00), whereas the median OS was 5.0 years for AdvSM (95% CI 2.12-NA) (Figure 3B). Moreover, as OS will always be confounded by age, we applied the age-matched propensity score matching (PSM) method to minimize selection bias, and found that the survival of patients with ISM was still favorable (*p* < 0.001) (Figure 3C).

**Figure 4:**
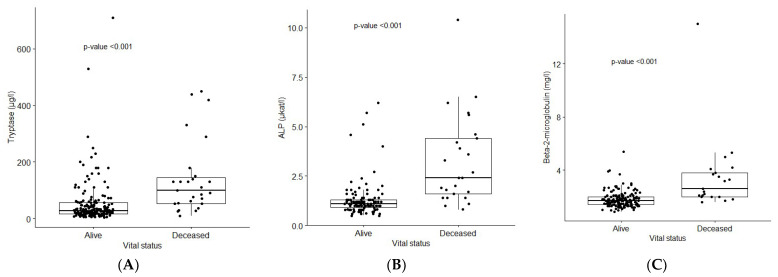
Associations between vital status and tryptase, ALP, and B2M levels: Boxplots were created to study relationships between vital status and (**A**) tryptase, (**B**) ALP and (**C**) B2M levels; *p*-values were calculated using the Mann–Whitney *U*-test.

Furthermore, we investigated the relationship between vital status and B2M, ALP, and sBT levels, and found significant increases in the levels of B2M (*p* < 0.001), ALP (*p* < 0.001), and sBT (*p* < 0.001) in non-survivors compared to survivors (Figure 4A–C).

When assessing parameters potentially affecting survival, we found that age at diagnosis, sBT, ALP, and B2M were significant in univariate analysis. However, when we applied a multivariate Cox regression analysis, increased ALP (HR 1.45; CI 1.08–1.96) and B2M (HR 1.5; CI 1.19–1.89) remained independent risk factors of overall mortality.

In our series, no ISM patients progressed to AdvSM. However, of 195 patients, 27 patients (13.8%) died during the follow-up period. Of the deceased patients, 11 had been diagnosed with ISM (including 1 with SSM), 10 had SM-AHN, and 6 had ASM. Causes of death in ISM patients were mainly due to other malignancies (55%) (three cases with GI cancer, one case with pancreatic cancer, one case with lung cancer, and one case with malignant melanoma), and in three cases were age-related. Of 10 cases with SM-AHN, the cause of death was due to the disease progression (n = 2) and intestinal ischemia (n = 1). In the remaining cases (two patients with ISM, seven with SM-AHN, and six with ASM), the cause of death was not obvious, although in some of the cases it was not possible to rule out SM-related death (Supplementary Table S2).

## 4. Discussion

In the present study, we describe our multidisciplinary experience at Karolinska University Hospital, which encompassed the care of SM patients for 15 years, starting in 2006. The strength of our cohort is that all patients are well-annotated, with a median follow-up of 7.5 years.

The reported frequency of SM variants may differ according to the type of center. In our multidisciplinary center, jointly led by allergology and hematology clinics, we found an overwhelming representation of patients with indolent SM (88.2%, including three patients with SSM). This is comparable to many previous studies, in which the frequency of ISM was reported to be 82% [10], 91% [11], and 91.2% [25]. Likewise, the frequency of AdvSM was less than 12% of cases in our cohort, which is consistent with the abovementioned single-center studies [10,11,25], and also similar to recently reported data from the European Competence Network on Mastocytosis (ECNM) registry (14.5%) [26]. Remarkably, however, this is much lower than the reported frequency of 54% in the Mayo Clinic study [27]. The discrepancies between these studies could be related to a selection bias, as some centers might be specialized in certain SM categories, e.g., the center in the Mayo Clinic mainly deals with patients with AdvSM. Moreover, patients with AdvSM were associated with older age in our series, but we could not confirm the previously reported difference regarding gender [11,28,29].

It is a well-known fact that the diagnosis of SM may often be delayed, as SM comprises a broad spectrum of signs and symptoms. Likewise, the median time for diagnostic delay was 10 years, with a broad range of 0.5–47 years, in our series. This observation is consistent with a previous Dutch study [25]. Interestingly, 26% of patients in our cohort had sBT levels below 20 ng/mL, and this may be one of the contributing factors for diagnostic delay. However, the most likely reason for diagnostic delay is the fact that patients with mastocytosis in the skin (MIS) have not been routinely investigated for systemic engagement in the past. Indeed, we had 45 patients (23%) with MIS in the cohort who had more than 15 years of diagnostic delay. Recently, raising awareness of the diagnosis and outcomes of systemic disease among dermatologists has enabled earlier diagnosis of SM.

The prevalence of SM in the adult population of the Stockholm region was 10.6 per 100,000 inhabitants during the study period. Likewise, we roughly estimated the mean incidence of new SM diagnosis/year in the Stockholm region, and found it to be 0.77 per 100,000 people per year. Our results are consistent with those of previous studies, such as a Dutch study that found a prevalence of ISM of about 13 per 100,000 adult inhabitants [9], and a Danish study that reported a prevalence of 9.59 cases per 100,000 inhabitants and an average incidence of 0.89 per 100,000 people per year [10]. In contrast, a recent Italian study reported relatively higher prevalence (17.2 per 100,000 adult inhabitants) and incidence (1.09 per 100,000 people per year) of SM in the adult population in the province of Verona [11]. However, the prevalence of SM in the Veneto region in the same study was 10.2 per 100,000 adult inhabitants, i.e., similar to our results [11]. Thus, there is a certain degree of consistency among European studies regarding the epidemiology of SM.

We found an overall prevalence of osteoporosis of 21.9%, and there were no significant differences among the different SM variants. This appears to be higher when compared to the 15% prevalence of osteoporosis in people over 50 years of age in Sweden [30]; however, this is consistent with a Dutch study reporting an overall prevalence of 22.4% among SM patients [25]. In contrast, this is less frequent compared to a French study showing a prevalence of 31% [31] and an Italian study showing a prevalence of 35.1% [11]. Although the underlying reasons are unclear, discrepancies between various studies may be partially due to dietary habits and/or related to physical activities. Furthermore, regarding osteosclerosis, all cases were associated with SSM and AdvSM variants, consistent with a previous observation [11]. This might indicate that the degree of BM MC infiltration may be of importance in the development of osteosclerosis in SM.

The overall frequency of comorbidities with solid tumors was 9.2% in our cohort. This is consistent with a previous study reporting an overall rate of 10% [9]. Interestingly, in our series, the most common type of solid tumor was cutaneous melanoma (2.5% of the total study cohort). All melanoma patients had concomitant skin lesions of mastocytosis. A potential association between SM and melanoma has previously been suggested [32,33]; however, further studies are needed to investigate the underlying mechanisms. Moreover, the cause of death was due to the solid tumors in 55% (6/11) of cases with ISM who died during the follow-up time.

The OS data of our cohort confirm the findings of previous studies showing that the patients with ISM have a favorable prognosis compared to AdvSM [26,27]. Furthermore, in the current series, two patients (both with ASM) received midostaurin (one for two months, and the other for four years at last follow-up—currently still ongoing). Naturally, it is difficult to determine whether midostaurin has contributed to the patient’s survival. Notably, although previous studies have demonstrated progression of ISM to advanced SM [4], we did not observe any progression from ISM to AdvSM in our cohort during the 15-year follow-up. Although we cannot assume that the limited follow-up time (median 7.5 years) was the only reason for not observing a progression from the indolent disease, it is worth considering that some of the reported cases in previous studies might have been incorrectly diagnosed at initial diagnosis. To support this notion, we should mention that two patients in our series were initially diagnosed with ISM but later deteriorated and developed SM-AHN. When we re-evaluated the patients’ original BM material, we noticed that these patients had indeed had SM-AHN from the beginning. Hence, this was not a disease progression, but a “misdiagnosis”. Hence, the correct classification of SM is important, because it serves as a major tool of prognostication.

We also evaluated the validity of certain prognostic factors, as several clinical and laboratory parameters have been used as prognostic variables for SM in previous studies [34,35,36,37,38]. Although in the univariable analysis, age at diagnosis and increased levels of tryptase, B2M, and ALP were all statistically significant prognostic factors for overall mortality, only B2M (HR 1.45, CI 1.08–1.96) and ALP (HR 1.50, CI 1.19–1.89) remained statistically significant prognostic factors in the multivariable analysis. Both ALP [35,39,40] and B2M [40,41,42] have already been shown to be of prognostic value for SM in previous studies, lending support to our findings. However, we did not check other suggested prognostic variables (e.g., multilineage involvement, variant allele frequency, and mutations in additional genes) in the present study, due to the limited numbers of patients with AdvSM.

Several limitations of this study should be addressed. Firstly, the retrospective design could lead to incomplete data. Furthermore, we should mention that the paucity of patients with AdvSM is another limitation, making generalizability difficult in the estimation of epidemiological measures. Additionally, in certain cases, we found it challenging to make the final diagnosis between SSM, ASM, and SM-AHN. In negative cases, where the clinical suspicion was strong, we either reassessed the biopsy material or performed a new BM biopsy yielding a different diagnosis. However, despite all of these efforts, the accuracy of final diagnosis in some cases may not have been 100%. Apparently, there are some traditional routines in different centers, but at our center, all patients with SSM or ASM, along with the majority of SM-AHN patients, are discussed at regular multidisciplinary conferences with the participation of allergists, hematologists, and pathologists (with those being the most experienced specialists at our center). In some cases, we also consult with other specialists, such as gastroenterologists, endocrinologists, or psychiatrists, depending on the individual patients’ symptomatology. Thus, a multidisciplinary approach may provide additional benefits in the care of those patients. On the other hand, the diagnosis of SM can be straightforward, and regular multidisciplinary meetings are optional in some other cases—for instance, patients with venom-induced anaphylaxis and indolent SM.

It should also be mentioned that our study encompasses the care of SM patients from 2006 to 2020. During this period, our knowledge has been evolved, as we have learned a lot about the presentation and diagnosis of this rare disease, and we have been following a standardized diagnostic workflow for SM patients in recent years. Thus, the patient care has also improved. Another major issue that we have noticed for correct diagnosis is the routine performance of flow cytometry analysis. For instance, identification of aberrant markers of CD2 and CD25 by flow cytometry can be essential, as this method is a more sensitive way of detecting these markers than immunohistochemistry. This would have important consequences, especially for patients with BMM, who usually do not have MC aggregates. Thus, if the presence of CD2/CD25 cannot be detected by immunohistochemistry, and if the patient has only two minor criteria, the patient would be misdiagnosed as not having SM. In addition, the *KIT* D816V mutation analysis of peripheral blood has been routinely performed in our center in recent years, and has become an important tool to help us to identify patients for BM evaluation—especially cases without skin lesions and without typical signs and symptoms of SM. More recently, we have also started analyzing the *KIT* D816V mutation allele burden, known as variant allele frequency.

## 5. Conclusions

In conclusion, the present study summarizes our 15 years of experience of SM, and underlines the need for standardized algorithms for diagnostic workup, risk stratification, and treatment. A thorough histological and immunohistochemical examination of the BM, along with flow cytometry analysis, is required for the correct diagnosis and classification of SM. In addition, our study confirms previous results on the prevalence and characteristics of SM, and expands on the notion that ISM with the major criterion has higher sBT levels than ISM without the major criterion, as well as being at higher risk of developing osteoporosis. Moreover, due to its heterogeneous nature and the complexity of the disease, the optimal care of patients with SM can be best managed in specialized centers using a multidisciplinary team approach.

## Figures and Tables

**Table 1:** Demographic and clinical characteristics of the enrolled subjects by variant of systemic mastocytosis at diagnosis.

Patient Characteristics	All Subjects	ISM	SSM	ASM	SM-AHN	*p*-Value ^#^	^¤^ Adj-*p*-Value
Number of subjects, n (%)	195	169 (86.6)	3 (1.6)	8 (4.1)	15 (7.7)	n/a	n/a
Age, median (range)	57.0 (20–84)	53.0 (20–84)	49 (48–60)	71 (53–84)	70.0 (49–83)	<0.001	<0.001
Male, n (%)	87 (44.6)	73 (43.2)	2 (66.7)	4 (50.0)	8 (53.3)	0.731	1.000
Skin engagement, n (%)	132 (67.7)	122 (72.1)	3 (100)	3 (37.5)	4 (26.6)	<0.001	<0.001
Major criterion, n (%)	112 (57.4)	89 (52.7)	3 (100)	8 (100)	12 (80.0)	0.019	0.285
Spindle-shaped MCs, n (%)	180 (92.3)	155 (91.7)	3 (100)	8 (100)	14 (93.3)	0.928	1.000
KIT D816V *, positive, n (%)	175 (96.7) * 14 n/a	150 (96.8) * 14 n/a	3 (100)	8 (100)	14 (93.3)	0.419	1.000
CD25/CD2 positive, n (%)	192 (98.5)	166 (98.2)	3 (100)	8 (100)	15 (100)	0.127	1.000
Tryptase µg/L, median (range)	32 (4.3–710)	27.5 (4.3–530]	450 (290–710)	140 (62–290)	130 (10–440)	<0.001	<0.001
ALP µkat/L, median (range)	1.2 (0.5–10.4)	1.1 (0.5–5.7)	4.0 (1.1–5.7)	4.1 (1.2–10.4)	2.3 (0.8–6.0)	<0.001	<0.001
ALP mg/L, ≥1.9 (%)	25 (18.0)	7 (6.1)	2 (66.7)	7 (88.0)	9 (66.7)	<0.001	<0.001
β2-Microglobulin mg/L, median (range)	1.8 (0.9–15.0)	1.7 (0.9–5.4)	2.4 (2.3–2.5)	3.1 (2.0–4.2)	3.5 (2.0–15.0)	<0.001	<0.001
β2-Microglobulin mg/L, ≥2.2, n (%)	37 (19.0)	20 (11.8)	1 (33.3)	5 (55.6)	11 (71.4)	<0.001	<0.001
Eosinophils > 0.5, median (range), n = 19	1.4 (0.5–14.8)	0.7 (0.5–1.5)	1.4 (1.4–1.4)	3.1 (0.6–5.3)	2.2 (1.2–14.8)	0.028	0.420
Monocytes > 0.8, median (range), n = 15	1.3 (0.9–5.5)	1.0 (0.9–1.7)	None	1.1 (1.1–3.2)	1.7 (1.30–5.5)	0.037	0.555
Osteoporosis **, n (%)	36 (21.9) 31 n/a	32 (22.2) 25 n/a	None	2 (28.5) 1 n/a	2 (20) 5 n/a	0.977	1.000

Abbreviations: ISM, indolent systemic mastocytosis; SSM, smoldering systemic mastocytosis; SM-AHN, systemic mastocytosis with an associated hematological neoplasm; ASM, aggressive systemic mastocytosis; MCs, mast cells; n/a, not analyzed. * Analyzed in 181 patients. ** Performed in 164 patients. ^¤^ Bonferroni-adjusted *p*-values. The new critical *p*-value was 0.003. ^#^ The group comparisons did not include patients with SSM, due to the paucity of patients, but compared ISM vs. ASM vs. SM-AHN.

## Data Availability

The data that support the findings of this study are not publicly available due to privacy or ethical restrictions. Further information about the data are however available from the authors upon reasonable request.

## Supplementary Materials

The following supporting information can be downloaded at: https://www.mdpi.com/article/10.3390/cancers14163942/s1: Please find supporting information regarding Tables S1 and S2. Table S1: Comparison of demographical, clinical and laboratory characteristics of ISM patients with or without major criteria at diagnosis. Table S2: Clinical characteristics of smouldering SM and aggressive SM patients.
