# Supplementary material for: Clinical Outcomes of Adults with Systemic Mastocytosis: A 15-Year Multidisciplinary Experience
Source: Cancers (Basel). 2022 Aug 16;14(16):3942. doi: 10.3390/cancers14163942 (PMC9405903; doi:10.3390/cancers14163942)
Supplement: Supplementary file 1 [file cancers-14-03942-s001.zip › cancers-1783931-supplementary.pdf]

SUPPLEMENTARY MATERIALS

Table S1. Comparison of demographical, clinical and laboratory characteristics of ISM patients with or without major criteria at diagnosis

Patient characteristics	All ISM subjects	ISM without major criterion	ISM with major criterion	*p*-value
Nr. of subjects, n	169	80	89	n/a
Age, median (range)	55 (20-84)	52 (20-83)	56 (22-84)	0.532
Male, n (%)	73 (43.2)	27 (33.8)	46 (51.7)	0.034
Skin engagement, n (%)	122 (72.1)	57 (71.2)	65 (73.0)	0.649
Spindle-shaped MCs, n (%)	155 (91.7)	75 (93.8)	80 (90)	0.064
*KIT D816V positive, n (%)	150 (96.8) *	74 (98.7)*	75 (93.8)*	0.55
CD25/CD2 positive, n (%)	167 (98.9)	79 (98.8)	88 (98.9)	NA
Tryptase µg/l, median (range)	27.5 (4.3-530)	20.5 (4.3-160.0)	46 (8-530)	<0.001
ALP µkat/l, median (IQR)	1.1 (0.5-5.7)	1.1 (0.5-2.0)	1.1 (0.6-5.7)	0.154
ALP mg/l, ≥1.9, n (%)	7 (6.1)	1 (2.0)	6 (9.5)	0.200
β2microglobulin mg/l, median (range)	1.7 (0.9-5.4)	1.7 (1.0-4.0)	1.7 (0.9-5.4)	0.851
β2microglobulin mg/l, ≥2.2, n (%)	20 (13.8)	9 (12.5)	11 (15.1)	0.836
Eosinophils >0.5, median (range), n = 8	0.7 (0.5, 1.5)	0.7 (0.5, 1.2)	1.1 (0.6, 1.5)	0.314
Monocytes >0.8, median (range), n = 5	1.0 (0.9, 1.7)	1.1 (1.0, 1.7)	0.9 (0.9, 1.7)	0.147
**Osteoporosis, n (%)	32 (22.2)	8 (11.6)	24 (32)	0.002

Abbreviations: ISM, indolent systemic mastocytosis; MCs, mast cells; n/a, not-analyzed.
*Analyzed in 155 patients. **Performed in 133 patients.

Table S2. Clinical characteristics of smouldering SM and aggressive SM patients

Patient N°, Gender	Diagnosis / year	B(1-3)* and C(1-5)** criteria	Treatment	Years of follow-up#
1, F	SSM/2003	B1, B2, B3	Corticosteroids, Oral cromolyn, H1-antihistamines, Hydroxyurea	13
2, M	SSM/2006	B1, B2	Corticosteroids, Oral cromolyn, α-interferon,	14
3, M	SSM/2010	B1, B2, B3	Corticosteroids, α-interferon	10
4, F	ASM/2014	C4, C5	Corticosteroids, Oral cromolyn, H1-antihistamines	2
5, M	ASM/2013	C1, C4, C5	Corticosteroids, Oral cromolyn, H1-antihistamines, Hydroxyurea,	7
6, M	ASM/2016	C1, C4, C5	Corticosteroids, Oral cromolyn, H1-antihistamines, Midostaurin	1
7, F	ASM/2014	B1, B2, C2, C3, C4	Midostaurin (for 4 years and still ongoing)	6
8, M	ASM/2015	B1, B2, C2, C4, C5	Corticosteroids, Oral cromolyn, H1-antihistamines	3
9, F	ASM/2015	B2, C4, C5	Corticosteroids, Oral cromolyn, α-interferon and Cladribine	4
10, M	ASM/2010	C1, C5	H1-antihistamines, Chemotherapy and Stem cell transplantation	5
11, F	ASM/2010	B2, C2, C4	Corticosteroids, Oral cromolyn, α-interferon	10

Abbreviations: SSM, smoldering systemic mastocytosis; ASM, aggressive systemic mastocytosis.

*B findings are: B1) High MCs burden on BM biopsy (>30% infiltration), serum tryptase >200 ng/ml; B2) signs of dysplasia or myeloproliferation with normal/slightly abnormal blood counts; B3) mild hepatosplenomegaly, and/or lymphadenopathy (>2 cm by Computerized Tomography or Ultrasound).
**C findings are more severe signs: C1) BM dysfunction with cytopenia, caused by MCs infiltration; C2) hepatosplenomegaly with ascites, elevated liver enzymes, portal hypertension; C3) splenomegaly with hypersplenism; C4) Malabsorption with hypalbuminemia and weight loss; C5) Large osteolytic lesions and/or severe osteoporosis with pathologic fractures.
#One patient died of lung cancer, one of unknown cause, and five of unspecified but likely of SM-related cause. Four patients are still alive.
